# Exoskeletons and Exosuits Could Benefit from Mode-Switching Body Interfaces That Loosen/Tighten to Improve Thermal Comfort

**DOI:** 10.3390/ijerph182413115

**Published:** 2021-12-12

**Authors:** Laura J. Elstub, Shimra J. Fine, Karl E. Zelik

**Affiliations:** 1 Department of Mechanical Engineering, Vanderbilt University, Nashville, TN 37212, USA; shimra.j.fine@vanderbilt.edu (S.J.F.); karl.zelik@vanderbilt.edu (K.E.Z.); 2 Department of Biomedical Engineering, Vanderbilt University, Nashville, TN 37212, USA; 3 Department of Physical Medicine & Rehabilitation, Vanderbilt University, Nashville, TN 37212, USA

**Keywords:** wearable technology, assistive device design, skin temperature, thermal acceptability, technology adoption

## Abstract

Exoskeletons and exosuits (exos) are wearable devices that physically assist movement. User comfort is critically important for societal adoption of exos. Thermal comfort (a person’s satisfaction with their thermal environment) represents a key design challenge. Exos must physically attach/interface to the body to apply forces, and these interfaces inevitably trap some heat. It is envisioned that thermal comfort could be improved by designing mode-switching exo interfaces that temporarily loosen around a body segment when assistive forces are not being applied. To inform exo design, a case series study (*N* = 4) based on single-subject design principles was performed. Our objective was to assess individual responses to skin temperature and thermal comfort during physical activity with a Loose leg-sleeve interface compared with a Form-Fitting one, and immediately after a Form-Fitting sleeve switched to Loose. Skin under the Loose sleeve was 2–3 °C (4–6 °F) cooler after 25 min of physical activity, and two of four participants reported the Loose sleeve improved their thermal comfort. After completion of the physical activity, the Form-Fitting sleeve was loosened, causing a 2–4 °C (3–8 °F) drop in skin temperature underneath for all participants, and two participants to report slightly improved thermal comfort. These findings confirmed that an exo that can quickly loosen its interface when assistance is not required—and re-tighten when it is— has the potential to enhance thermal comfort for some individuals and environments. More broadly, this study demonstrates that mode-switching mechanisms in exos can do more than adjust physical assistance: they can also exploit thermodynamics and facilitate thermoregulation in a way that enhances comfort for exo users.

## 1. Introduction

Exoskeletons and exosuits (exos) are defined as wearable devices that augment, enable, assist, or enhance motion, posture, or physical activity [1]. Exos biomechanically assist tasks ranging from bending, lifting, and reaching [2,3] to walking, running, and jumping [4]. Exos are used in a range of different applications, such as clinical [5] and occupational [6]. Exo research has primarily focused on device design and validation that exos reduce muscular demands, effort, or fatigue [7,8]. Despite the focus on exos as physical assistance devices, frequently the key barriers to societal adoption are related to comfort, fit, and freedom of movement [9]. Recent studies have begun to assess physical comfort thresholds when exo forces are applied to different parts of the body, identifying that comfort limits increase with habituation [10] and padded materials [11]. In addition, static, dynamic, and cognitive fit characteristics have been defined for consideration during exo development and evaluation [12]. 

However, one area that has received little attention from the exo research community is thermal comfort, which is defined as a person’s satisfaction with their thermal environment [13]. Thermal comfort is influenced by thermodynamics (the physics of heat and temperature) and thermoregulation (the process by which the body maintains its temperature). While there is some published literature documenting the issue of thermal comfort with exo use (e.g., [13,14]), the most compelling motivation comes directly from exo early adopters in industry, who have commonly reported thermal comfort as a barrier to user adoption [15,16].

Maintaining thermal comfort is challenging since exos must attach to the body to apply assistive forces, and these physical body interfaces (termed interfaces) inevitably trap some amount of heat [14]. Heat retention is due, in part, to the lack of space between the skin and interface which inhibits airflow, the transfer of heat generated by the body, and evaporation of sweat to the surrounding environment. Similar to other wearable accessories (e.g., backpacks, performance apparel), thermal comfort can be partially improved through material selection and design [17]. For instance, minimizing the size of the interface can reduce the surface area where heat is retained. However, if an interface (e.g., sleeve, cuff) is too small or thin, it can become physically uncomfortable under load due to pressure points on the skin, or too structurally weak to transmit exo forces to the body. Consequently, exo interface design aims to balance thermal and physical comfort with adequate material strength to apply requisite forces. 

It is envisioned that the thermal comfort of exo interfaces could be improved—without degrading physical comfort or structural integrity—by creating a mode-switching mechanism that can loosen and tighten the interface around a body segment. While mode-switching designs have been implemented to turn on/off or adjust physical assistance (e.g., [2,3,4,9]), this concept has not yet been applied to enhance the breathability or thermal comfort of exos. Dual-mode exo interfaces could provide an effective and practical way to enhance thermal comfort, since most exos only provide intermittent assistance, rather than continuous. An interface could be loosened when an exo is not providing assistive forces, and subsequently re-tightened onto a body segment before assistance is required. The precise exo implementation, actuation, power source, and frequency of mode-switching would depend on the specific end-user and application, but various technical designs have been conceived and prototyped for creating and controlling this dual-mode interface function [18] in a way that is quick, practical, and easy to use.

Before refining and optimizing new dual-mode (loosening/tightening) interfaces, it was important to evaluate the difference a loosened sleeve would make to users relative to a conventional form-fitting exo interface. The objective of this study was to assess individual responses to skin temperature and thermal comfort during physical activity with a loose leg-sleeve compared with a form-fitting one, and immediately after a form-fitting sleeve switched to loose mode. For the purpose of this study, the dual-mode leg sleeve was designed for integration with a back-assist exo [3,7]. However, it is expected that this dual-mode concept would also be a viable solution for interfacing with other body segments and other types of exos. These results have informed our own interface design and are being shared as the findings and design approach may be valuable to other exo developers exploring ways to improve thermal comfort. Additionally, the concept of using mode-switching behaviors not only to adjust physical assistance, but also to modulate thermodynamics and thermal comfort, represents a paradigm shift that could enhance user experience and help accelerate societal adoption of exos. Stated more broadly: while current mode-switching exos successfully leverage one subfield of mechanics (classical mechanics) to adjust the level of physical assistance, there are promising opportunities to exploit or integrate other sub-fields of mechanics (e.g., thermodynamics) to further improve user experience.

## 2. Materials and Methods

### 2.1. Participants

In this study, four male participants (Height: 1.7–1.9 m, Mass: 65–88 kg, Age: 23–35 years) who exercised regularly and had no history of injury in the past six months prior to testing, provided informed written consent following ethical approval from the Institutional Review Board (Vanderbilt University). This experiment uses a single-subject study design, which typically only includes 1–3 participants [19], since each participant serves as their own control. The study was performed at an ambient temperature of 22.7 ± 0.5 °C and humidity of 40.5 ± 1.5%, and participants were exposed to this environment for thirty minutes before testing [20].

### 2.2. Leg Sleeve Interfaces

**Figure 1:**
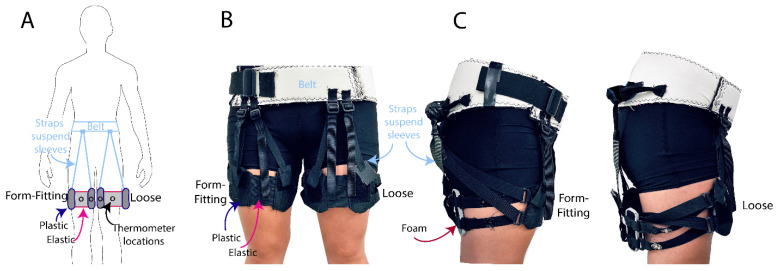
Leg sleeve interfaces in Loose and Form-Fitting modes. (**A**) Schematic of the mode-switching interface design. The symbol ᴼ represents the location of the four thermometers, placed underneath the plastic and elastic components of each leg sleeve during experimental testing. Photographs showing a (**B**) frontal view and (**C**) side view of the Loose and Form-Fitting sleeves.

Leg sleeves (Figure 1) were constructed from materials commonly used in commercial exo interfaces, including 0.93-inch-thick plastic (Kydex thermoplastic), 1/8 inch EVA foam, 0.5–1.5 inch wide flat nylon webbing and fabric elastic, and commercial off-the-shelf plastic buckles. Each sleeve weighed approximately 0.2 kg. The leg sleeves were adjusted for each participant individually, and worn with no exo actuation components (e.g., springs, motors) to avoid confounding factors. These functional prototype sleeves loosen and tighten using a simple strap adjuster mechanism, similar to backpack straps [18], although there are many alternative ways to incorporate the loosening/tightening capability, and the specific mechanism used was not integral to the thermal comfort effects assessed in this study. Leg sleeves were positioned on the lower or middle third of each thigh, a common interface location for upper- and lower-body exos. Webbing straps were connected between the sleeves and a waist belt, to suspend the sleeves when loosened and prevent them from slipping down the leg.

### 2.3. Skin Temperature

Skin temperature was measured using four FDA-approved thermometers with accuracy to ± 0.1 °C ([21], VAVA Model: VA-IH008). These thermometers were easy to sanitize and allowed remote monitoring during the COVID-19 pandemic. Two thermometers were placed on the anterior surface of each thigh (directly on the skin), with one under the elastic and one under the plastic portion of each leg sleeve (Figure 1) to monitor temperature changes under two different materials commonly used in exo interfaces. Skin temperature was chosen for use, as it is closely related to thermal sensation and comfort [22]. 

### 2.4. Subjective Thermal Ratings

Subjective thermal ratings of each leg sleeve were assessed using methods recommended by Schweiker et al. [23]. Thermal comfort, which reflects someone’s satisfaction with their thermal environment [24], was rated on a 6-point scale from ‘very uncomfortable’ to ‘very comfortable’ [25]. In this context, the environment refers to the leg under each sleeve. Thermal sensation, which reflects how warm someone feels, was rated on an 8-point scale from ‘very cold’ to ‘very hot,’ [24]. Thermal acceptability reflects someone’s willingness to tolerate their thermal environment and was rated on a 4-point scale from ‘unacceptable’ to ‘acceptable’. Participants were also invited to provide additional verbal feedback.

### 2.5. Protocol

This protocol was developed based on single-subject study design principles [7,19]. Each participant served as their own control by comparing a Loose sleeve on one leg vs. a Form-Fitting sleeve on the other, and then switching from a Form-Fitting to Loose sleeve to characterize the effects of switching modes. Participants donned two leg sleeves: one affixed snugly around the right leg (Form-Fitting condition), while the other was loosened around the left leg (Loose condition). The Form-Fitting sleeve was worn flush against the skin, similar to Spandex leggings, and similar to how commercial exo thigh sleeves are commonly worn. The Loose sleeve fit like shorts, with an approximately 5 cm greater circumference than the participant’s thigh. Three initial skin temperature and perceived thermal comfort ratings were recorded immediately after donning each leg sleeve, to capture the baseline mean and standard deviation around this baseline measurement. Participants then performed a series of consecutive physical activities without a rest period, where walking was performed on a split-belt, force-instrumented treadmill (Bertec, Columbus, USA): (i) five minutes of treadmill walking at 1.3 m/s (slower walking), (ii) five minutes of treadmill walking at 1.6 m/s (faster walking), (iii) fifteen minutes of manual handling tasks (lifting 5 and 10 kg boxes and carrying them over 5 m). Walking speeds were chosen based on a recent meta-analysis which identified ‘usual’ walking pace as 1.3 m/s and moderate to fast walking as 1.5–1.7 m/s [26]. Lift and carry tasks were a subset of those incorporated in previous research, representative of tasks commonly performed by manual material handlers [27]. Collectively, these were chosen as simple, repeatable tasks of moderate physical activity, though many other tasks could have also been used.

Skin temperature and thermal ratings were recorded upon completion of each activity. Next, the Form-Fitting sleeve was loosened following completion of the lifting/carrying tasks, and participants walked on the treadmill for an additional five minutes at 1.3 m/s. Skin temperature and thermal ratings were recorded again, to assess the effect of switching the leg sleeve from Form-Fitting to Loose.

### 2.6. Analysis

Owing to the subjective and individual nature of perceived comfort, particularly for exos [7,10], data were quantified and analyzed on an individual basis following single-subject research design principles [28]. Subject-specific skin temperature (°C) and thermal ratings were plotted. Readings from the two thermometers on each leg were averaged for each participant to obtain a single temperature value per leg sleeve condition. The absolute difference between Form-Fitting and Loose sleeves was calculated for each participant to allow comparison between conditions. Within-participant comparisons were used to identify trends in skin temperature or thermal ratings (i) during physical activity when the sleeve was Loose vs. Form-Fitting, and (ii) immediately after the sleeve transitioned from Form-Fitting to Loose. We received verbal feedback from all participants, and this was analyzed using thematic analysis following Braun and Clarke’s 6-step framework [29]. The identified themes were used to support and better understand results from skin temperature data and subjective ratings.

## 3. Results

**Figure 2:**
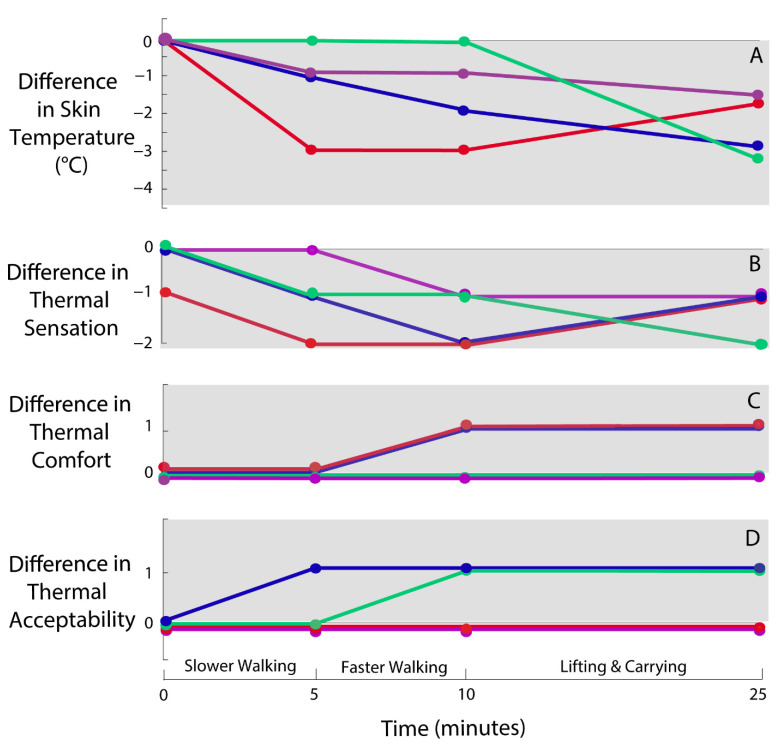
Differences in skin temperature and thermal ratings between Loose and Form-Fitting conditions during physical activities. Each color represents one participant. Each dot designates when in the experimental protocol a measurement was taken. Lines were added to easily visualize individual participant trends. Values on the gray background represent improvements in skin temperature (**A**), thermal sensation (**B**), thermal comfort (**C**), and thermal acceptability (**D**) for the Loose sleeve relative to the Form-Fitting sleeve. In each plot, a value of zero signifies no difference between the Loose vs. Form-Fitting sleeve.

For all participants, skin temperature was lower in the Loose sleeve compared with the Form-Fitting sleeve during/after physical activity (Figure 2A). Skin temperature was on average 1 °C (range: 0–3 °C) lower during slower walking, 1 °C (0–3 °C) lower during faster walking, and 3 °C (2–3 °C) lower during lifting/carrying. These changes in skin temperature were much larger than the standard deviations in baseline skin temperature recordings, which ranged from 0–0.3 °C across participants. Thermal sensation results followed a similar trend when comparing Loose vs. Form-Fitting (Figure 2B, Table 1 and Table 2). The Form-Fitting sleeve changed from ‘neutral’ at the start of the protocol to either ‘slightly warm’ or ‘warm’ by the end of the physical activities, whereas the Loose sleeve remained ‘neutral’ throughout (Figure 2B).

Thermal comfort and acceptability trends (Figure 2C,D, Table 3, Table 4, Table 5 and Table 6) were subject-specific: two participants reported slight improvements (1-point on each scale) in favor of the Loose sleeve, whereas two participants reported no differences between the Form-Fitting and Loose sleeves. For instance, with the Loose sleeve, all participants rated thermal comfort as ‘neutral’ or ‘comfortable’ throughout the protocol. However, with the Form-Fitting sleeve, only two participants reported ‘neutral’ throughout, while the other two reported ‘slightly uncomfortable’ or ‘slightly comfortable’ after physical activity (Figure 2C).

**Figure 3:**
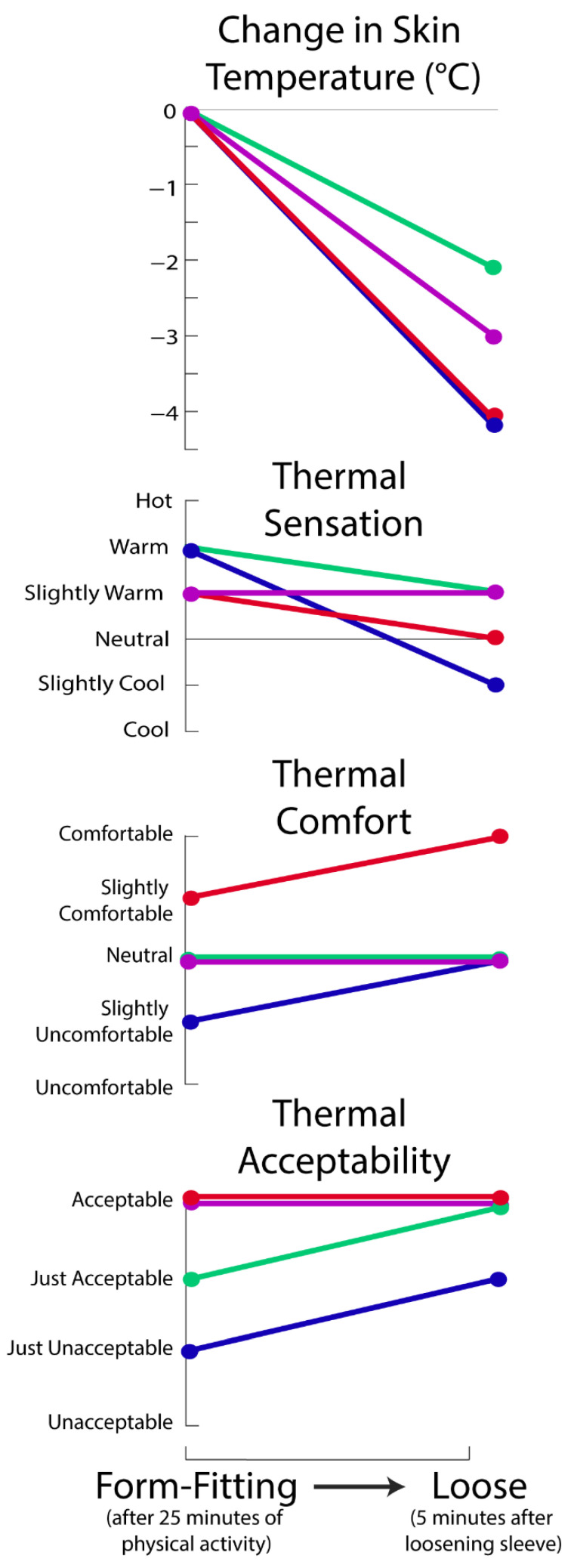
Skin temperature and thermal ratings after the Form-Fitting sleeve had been worn for 25 min of physical activity, and then five minutes after it was loosened. Each color represents one participant. Lines between time-points are for ease of visualizing. Subject-specific data are also reported in Table 1, Table 2, Table 3, Table 4, Table 5 and Table 6.

When the Form-Fitting sleeve was switched to Loose at the end of the protocol (Figure 3), there was a decrease in skin temperature for all participants (average: 3 °C, range: 2–4 °C). Overall, three of four participants reported a cooler thermal sensation, and two of four reported improvements in thermal comfort and acceptability after the Form-Fitting sleeve was loosened (Figure 3, Table 1, Table 2, Table 3, Table 4, Table 5 and Table 6).

## 4. Discussion

Two key themes were identified from the results: Firstly, the Loose sleeve was cooler and perceived to be cooler during physical activity. Half of the participants reported that the Loose sleeve led to improvements in their thermal comfort or acceptability relative to the Form-Fitting sleeve. Secondly, all participants exhibited a reduction in skin temperature when the Form-Fitting sleeve was loosened after physical activity, and most participants (three of four) felt this resulted in their leg feeling cooler under the sleeve. Moreover, half the participants reported the transition from Form-Fitting to Loose led to improvements in their thermal comfort or acceptability following physical activity. These findings confirm that, at least for a subset of individuals and environments, an exo that can loosen its interface to the body when assistance is not needed has the potential to reduce heat build-up on the skin and improve thermal comfort. 

Mode-switching interfaces could be beneficial during prolonged periods or for brief interface loosening breaks (e.g., on the order of seconds or minutes). Air circulation or ventilation within clothing reduces insulation [30]. Air circulation over the skin is affected by tightness of clothing, and a looser fit allows evaporative cooling to occur, carrying off excess heat, consequently maintaining skin temperature and thermal comfort [31]. Therefore, it appears the Loose sleeve provides sufficient space between the skin and sleeve, enabling improved transfer of heat to occur [17] and resulting in a cooler skin temperature compared with the Form-Fitting sleeve. The ability to quickly switch from a Form-Fitting to Loose sleeve during rest breaks or tasks when assistance is not needed could therefore be a valuable feature for users to improve thermal comfort. 

The results of this study, in combination with prior literature, suggest that mode-switching interfaces provide a promising solution to improve thermal comfort while wearing exos in temperate or warm environments. Skin temperature naturally cools down during physical activity [32], and hot skin temperatures (>35 °C) have been found to impair submaximal aerobic performance [33]. A decrease in skin temperature was observed with the Loose sleeve. However, with the Form-Fitting sleeve, the skin temperature underneath remained stable or slightly increased (0–2 °C change) throughout the physical activities. This indicates that the Form-Fitting sleeve inhibited the transfer of heat, and likely also the evaporation of sweat, to the surrounding environment [17,34] relative to the Loose sleeve. For two participants, the Form-Fitting sleeve also resulted in a decrease in thermal comfort and acceptability (Figure 2). All participants experienced a warmer thermal sensation with the Form-Fitting sleeve during physical activities. In addition, since thermal discomfort is related to skin sweating [35], it is possible that for some individuals a decrease in thermal comfort only occurs with tasks of longer duration, or when undertaking more demanding activities which cause increased sweat production [36]. These results are supported by Liu et al. (2021), who also found an increase in mean skin temperature and thermal sensation during a 20-min lifting task with a back-assist exo in both cold (10 °C) and temperate (21 °C) environments. Interestingly, while Liu et al. (2021) found the exo to reduce thermal comfort in the temperate environment, they found thermal comfort and sweating were substantially improved by wearing the exo in the cold environment. Thus, the main benefits of mode-switching body interfaces may be for exos worn in temperate or warm environments. 

Once the transition from Form-Fitting to Loose occurred, heat was released, and participants perceived a cooling effect on their leg. The results were reinforced by subjective evaluation and verbal feedback from participants. For instance, when switching from Form-Fitting to Loose, participants reported they ‘immediately felt as though there was a big change in temperature’, ‘[their skin] felt cold immediately after it loosened’, and ‘the change in temperature was instant and felt dramatic.’ The combined results (Loose vs. Form-Fitting, and the transition from Form-Fitting to Loose) demonstrate complementary ways a mode-switching exo interface can be effective, first by maintaining a lower skin temperature during longer periods of physical activity when assistance is not required, and secondly by providing an immediate reduction in thermal sensation and skin temperature when the sleeve is loosened. Collectively, skin temperature trends, thermal ratings, and verbal feedback suggest that a dual-mode exo interface (e.g., loosening/tightening leg sleeve) has the potential to meaningfully improve thermal comfort and acceptability for a subset of users. 

Nevertheless, there are limitations to acknowledge. This was a case series conducted on a subset of tasks in a particular ambient environment. The sample size of four is small, but was appropriate for this type of single-subject protocol [19]. Average temperature values were reported for each leg for brevity, but temperature measurements and trends at individual thermometer locations matched those observed when averaging over each leg. The interfaces in this study were directly on the skin and tested on the legs. However, we expect similar results and trends for exo interfaces worn over clothing based on informal testing and other common life experiences. For instance, after wearing a backpack over a shirt for a prolonged period, momentarily lifting the backpack off the shirt provides noticeable thermal relief. A rare exception may be if the clothing underneath is already very tight and thermally insulated, such that changes to an exo interface worn externally may have limited effect on thermal comfort. The mode-switching mechanism was designed to provide thermal relief during short or prolonged breaks, not to continuously change tightness/looseness during dynamic movements, which would require a motorized system to make sub-second adjustments. The relative benefits of more complex (e.g., motorized, automated) mode-switching mechanisms would need to be explored relative to the thermal comfort value they provide to a given end user.

This study presented data from male participants only; however, pilot testing was performed on two female participants who demonstrated the same trends as those presented in Figure 2 and Figure 3. For instance, both female pilot participants exhibited an increase in skin temperature following 10 min of self-paced walking with the Form-Fitting sleeve, followed by a decrease in skin temperature when the Form-Fitting sleeve was loosened. These additional methods and data were omitted for brevity due to differences in the pilot protocol (e.g., order and duration of physical activities), and because they did not alter conclusions. It is expected that loosening exo interfaces to reduce trapped heat can improve thermal comfort for users of different sexes, ages, races, and ethnicities. In addition, similar trends are also expected as in Figure 2 and Figure 3 if interfaces were worn over trousers, or if temperature was measured on additional areas of skin under the sleeves, or if interfaces had been worn on other body segments. However, thermal comfort varies between individuals and is affected by other environmental factors (e.g., air temperature and speed [37]) and personal factors (e.g., metabolic rate and clothing insulation [37]), as well as the duration of physical activity and exo use [38]; which is why it was not surprising to see some inter-subject differences in thermal survey ratings. 

This study was primarily intended to inform our own design work and was sufficient for our purposes; however, the data, general observations, and innovative design approach may also be of value to other exo developers or those looking to build upon this research. Specifically, this study represents a shift in how insights from biomechanics (the study and application of mechanical principles applied to biological systems) can be used to drive innovations in exo development. To date, the design of mode-switching exos has focused on applying principles from classical mechanics to reduce musculoskeletal demands, enhance physical performance, and avoid movement restrictions. However, this study highlights how other sub-fields of mechanics (e.g., thermodynamics) can also be leveraged within mode-switching exos to positively impact the comfort and experience of exo users. 

Exploiting classical mechanics principles (e.g., leverage) to assist or augment biological movement, in conjunction with principles of thermodynamics to improve thermal comfort, has the potential to improve user acceptance of and benefits from exos. Not only has thermal discomfort been identified as a barrier to exo use [15,16], it has also been shown to affect productivity [39,40]. Improving thermal comfort therefore has the potential to increase exo adoption and impact. In clinical settings, this could result in more individuals with disabilities benefitting from assistive or rehabilitative exos. In occupational settings, exos have been shown to decrease muscle activity and fatigue [6,41], thus increased exo adoption could reduce the risk of overuse injury, as well as increase productivity. Ergonomic assessment tools (e.g., [42]) suggest that, under various conditions, it is possible to simultaneously reduce injury risk and increase productivity (e.g., number of lifting repetitions) when wearing an exo. This highlights how exos fit within and contribute to emerging trends, such as sustainable work and industry 5.0, which place the long-term wellbeing of the worker at the center of the process, and leverage collaboration between humans and machines to benefit industry, workers, and society.

Future exo research on thermal comfort should examine longer duration tasks, under different environmental conditions (e.g., hot and cold), with a greater number and diversity of participants and with mode-switching interfaces on different body parts. To ensure representativeness and generalizability, the specific details of future protocols, such as duration of tasks, should be determined by the research question and intended use case and population. A further area for consideration would be to evaluate the influence of material selection in exo interface design or the use of sweat minimizing materials for clothing in conjunction with the proposed mode-switching, loosening/tightening interfaces, and their combined impact on thermal comfort of the user. In addition, research here evaluated the exo interfaces in isolation. Future studies should integrate mode-switching interfaces into fully functioning exos, then test them on intended user groups to understand behavioral aspects, such as how often mode-switching is used, and to assess thermal comfort benefits in realistic/operational environments. The Human Readiness Level Scale developed by the Human Factors and Ergonomics Society outlines a progression from basic research and development of systems to full-scale validation and verification [43]. These guidelines could be used to design a progression of larger controlled studies on thermal comfort of exos, followed by large-scale experiments performed in operational environments with intended end users. Of note, industrial standards for exos are also being developed by organizations, such as the ASTM International Standards Committee, to propose test methods for evaluating various aspects and effects of exos such as on user comfort.

## 5. Conclusions

This study highlights the importance of the design and optimization of physical interfaces between the human body and wearable assist devices. We found that the skin under the Loose sleeve was 2–3 °C (4–6 °F) cooler after 25 min of physical activity, and two of four participants reported the Loose sleeve improved their thermal comfort. After completion of the physical activity, the Form-Fitting sleeve was loosened, causing a 2–4 °C (3–8 °F) drop in skin temperature underneath for all participants, and two participants to report slightly improved thermal comfort. These findings suggest that an exo that can loosen its interface when assistance is not required—and re-tighten when it is—has the potential to enhance user comfort, during both prolonged and brief loosening periods.

## Figures and Tables

**Table 1:** Thermal Sensation results for each participant for the Form-Fitting Leg Sleeve, where −4 represents very cold, −3 cold, −2 cool, −1 slightly cool, 0 neutral, 1 slightly warm, 2 warm, 3 hot, and 4 very hot.

	Participant 1	Participant 2	Participant 3	Participant 4
Baseline	0	1	0	0
Slower walking	2	1	1	0
Faster walking	3	1	1	1
Lifting and carrying	2	1	2	1
Slower walking (five minutes after loosening sleeve)	−1	0	1	1

**Table 2:** Thermal Sensation results for each participant for the Loose Leg Sleeve, where −4 represents very cold, −3 cold, −2 cool, −1 slightly cool, 0 neutral, 1 slightly warm, 2 warm, 3 hot, and 4 very hot.

	Participant 1	Participant 2	Participant 3	Participant 4
Baseline	0	0	0	0
Slower walking	1	−1	0	0
Faster walking	1	−1	0	0
Lifting and carrying	1	0	0	0

**Table 3:** Thermal Comfort results for each participant for the Form-Fitting Leg Sleeve, where −3 represents very uncomfortable, −2 slightly uncomfortable, −1 uncomfortable, 0 neutral, 1 slightly comfortable, 2 comfortable, and 3 very comfortable.

	Participant 1	Participant 2	Participant 3	Participant 4
Baseline	0	2	0	0
Slower walking	1	2	0	0
Faster walking	−1	1	0	0
Lifting and carrying	−1	1	0	0
Slower walking (five minutes after loosening sleeve)	0	2	0	0

**Table 4:** Thermal Comfort results for each participant for the Loose Leg Sleeve, where −3 represents very uncomfortable, −2 slightly uncomfortable, −1 uncomfortable, 0 neutral, 1 slightly comfortable, 2 comfortable, and 3 very comfortable.

	Participant 1	Participant 2	Participant 3	Participant 4
Baseline	0	2	0	0
Slower walking	1	2	0	0
Faster walking	0	2	0	0
Lifting and carrying	0	2	0	0

**Table 5:** Thermal Acceptability results for each participant for the Form-Fitting Leg Sleeve, where −1 represents unacceptable, 0 just unacceptable, 1 just acceptable, and 2 acceptable.

	Participant 1	Participant 2	Participant 3	Participant 4
Baseline	1	2	2	2
Slower walking	0	2	2	2
Faster walking	0	2	1	2
Lifting and carrying	0	2	1	2
Slower walking (five minutes after loosening sleeve)	1	2	2	2

**Table 6:** Thermal Acceptability results for each participant for the Loose Leg Sleeve, where −1 represents unacceptable, 0 just unacceptable, 1 just acceptable, and 2 acceptable.

	Participant 1	Participant 2	Participant 3	Participant 4
Baseline	1	2	2	2
Slower walking	1	2	2	2
Faster walking	1	2	2	2
Lifting and carrying	1	2	2	2
